# Cross-Cultural Adaptation of Research Tools: A Study on the Cultural Intelligence Scale Adaptation in Slovenian

**DOI:** 10.5964/ejop.v14i2.1527

**Published:** 2018-06-19

**Authors:** Eva Boštjančič, R. Boyd Johnson, Urša Belak

**Affiliations:** a Department of Psychology, Faculty of Arts, University of Ljubljana, Ljubljana, Slovenia; b Indiana Wesleyan University, Marion, IN, USA; Webster University Geneva, Geneva, Switzerland; University of Bari "A. Moro", Bari, Italy

**Keywords:** adaptation, cultural intelligence, cross-cultural communication, cross-cultural interaction, bias

## Abstract

The article examines the cross-cultural transferability of a widely accepted cross-cultural assessment tool – the Cultural Intelligence Scale (CQS) – using research conducted in Slovenia and insights from the American and Slovenian researchers who translated the tool into Slovene and adapted it for use in Slovenia. In the context of a qualitative focus group based study, the researchers look at the specific characteristics of CQS perceptions within the Slovenian sample (two focus groups – one in the capital and the other in the south of the country) and identify barriers to these perceptions and the specific characteristics of the perceptions of Slovenian citizens regarding cross-cultural interaction.

## The Importance of Cultural Intelligence and Testing the Cross-Cultural Transferability of the CQS

Increasing globalisation and the mixing of cultures in various fields is leading to an increased focus on the ability to work effectively in an intercultural environment. In the scientific context, this manifests itself as a need for the systematic study of cultural intelligence, a concept that made its first appearance in 2003 and which is defined as "an individual's capability to function and manage effectively in culturally diverse settings" (Ang & Van Dyne, 2008a, p. 3). In order to research this concept, a Cultural Intelligence Scale (CQS; Ang & Van Dyne, 2008b) has been developed to measure the four primary factors which represent distinct cultural intelligence’ capabilities: drive, knowledge, strategy and action. It is a 20-item, four-factor scale. Ang et al. (2007) state that cultural intelligence examines specific spheres in intercultural settings. This multidimensional construct includes four dimensions of cultural intelligence:

- cognitive – "an individual's cultural knowledge of norms, practices and conventions in different cultural settings" (Van Dyne, Ang, & Koh, 2008, p. 16),
- metacognitive – "an individual's cultural consciousness and awareness during interactions with those from different cultural backgrounds" (Van Dyne et al., 2008, p. 16),
- motivational – "an individual's capability to direct attention and energy toward cultural differences" (Van Dyne et al., 2008, p. 16), and
- behavioural – "an individual's capability to exhibit appropriate verbal and non-verbal actions when interacting with people from different cultural backgrounds" (Van Dyne et al., 2008, p. 16).

While some researchers assume that the surveys can be used anywhere, irrespective of where they were developed, others (e.g. Goh, 2009) warn that the concepts and theories are only transferable in cultures where cultural norms and values are similar. The purpose of adaptation is to better fit the needs of a new population, location language, or mood, or any combination of these (Harkness, 2008; Paulston & Tucker, 2003). To narrow the gap of Western assessments being used in Eastern cultures (Barnes, Buko, Johnson, & Kostenko, 2012), this study is seeking through qualitative and quantitative research to determine what language must be used to ensure these.

Besides the adequacy of the translation of the CQS into Slovene, we were interested in how Slovenes understand the cross-cultural interaction and cultural intelligence and their attitude towards the latter. This, in fact, provides us with an insight into how people view the concept itself.

Two focus groups were organised in two municipalities where development is above average – the capital city Ljubljana and Koper in the south-western corner of the country, representing a rural environment. As the capital city, Ljubljana represents a highly intercultural diverse centre in the middle of the country, while Koper is a coastal town that forms part of a bilingual region since it lies very close to the border with Italy.

As already highlighted by Goh (2009), the transferability of the tool depends on cultural values and norms. Understanding of the CQS and the individual items in it is affected by a range of geographical, ideological, social, cultural and psychological factors. Below, then, we set out some of the characteristics of Slovenian culture that could potentially influence the perception of individual respondents.

## Demographics of Slovenia and Diversity Statistics

As a small country with a population of just over two million (2.063.371) (Statistični urad Republike Slovenije [SURS], 2016a), Slovenia represents an interesting example in terms of the mixing of different cultures. Today the country is increasingly open to foreigners, both migrants and tourists, and a consequence of this is growing contact between Slovenes and other cultures. On the other hand, a growing trend of people leaving the country to go abroad – be it because of economic conditions, to seek a better standard of living, or tourism – may be observed. Slovenia's strategic position at the heart of Europe, at a point of contact between various worlds, also plays its part. One of the smallest countries of the European Union (20.273 km^2^; SURS, 2016a), Slovenia is also a transit country. The official and state language is Slovene, while in the areas in which the Italian and Hungarian national minorities are concentrated, Italian and Hungarian respectively are also official languages.

The percentage of foreign nationals in the population is 5.3%, which means that there are currently 104.197 foreigners living in Slovenia (SURS, 2016b). Further statistics show that 15.420 people arrived from other countries in 2015, while 14.913 people left the country. This indicates positive net migration. The reasons for immigration vary (SURS, 2016b):

- favourable economic conditions and increased demand for workers which in certain sectors (e.g. construction) Slovenia's labour market was unable to meet;
- Slovenia's entry to the European Union (EU), resulting in an increase in immigration to Slovenia by citizens of some new EU member states;
- family reunification (secondary immigration of family members of foreign nationals already resident in Slovenia for whom Slovenia has become an immigration target country).

Women account for 35% of foreign nationals and thus represent 3.4% of the total female population of Slovenia. The larger share of male foreign nationals is the consequence of economic migrations. The most numerous groups among other nationalities in Slovenia are citizens of the former Yugoslavia, headed by citizens of Bosnia and Herzegovina (53.681) (RS, Ministrstvo za notranje zadeve, 2016) and followed by those from Kosovo, Serbia, Macedonia and Croatia. These are economically less developed countries whose citizens frequently come to Slovenia as a foreign workforce (recent economic immigrants from the countries of the former Yugoslavia, in particular Kosovo and Macedonia). These immigrants are predominantly men (around 75%). Since Slovenia was part of a federal state with these nations 25 years ago, many migrations are also the consequence of the fact that families were forcibly separated by the new borders that appeared following the break-up of the country. This means that when they move to live with relatives they are no longer moving from one end of the country to the other, but migrating from one country to another. The second wave of immigrants from this region is represented by refugees from war zones (Bosnia and Herzegovina) (SURS, 2016c). Citizens of "third countries", i.e. countries that are not EU members, are strongly prevalent among foreign nationals in Slovenia (81%), and in most cases these are citizens from the countries of the former Yugoslavia. The largest number of foreign nationals from EU countries in Slovenia are from Croatia, followed by Bulgarians, Italians, Germans and Slovaks.

## Impact of Other Cultures and Regional Peculiarities of Slovenia

In the past Slovenia was an area of intersection between the Germanic world to the north, Italian influences to the West and the Balkans to the south. These different influences have rendered more complex the task of shaping a uniform national identity and language. The national borders have changed frequently (which in practice means that Slovenia has grown smaller, particularly in the north). Slovenia's population has shown a constant fall, particularly in the twentieth century, for a variety of reasons, most notably economic migrations and the Fascist dictatorship (1941-1945). The political boundaries have never corresponded exactly to national (ethnic) boundaries.

Despite Slovenia's small size, considerable regional, spatial and economic divisions may be observed in the country. Western and central Slovenia have an above-average economic structure that is interrupted by individual belts of below-average development (Kušar & Nared, 2004). By contrast, large parts of eastern Slovenia are characterised by strongly below-average economic structures, pointing on the one hand to a division into developed urban centres where the majority of economic activities are concentrated and, on the other, to social erosion that has mostly affected remote areas, in particular border areas and rural areas (Pečar, 2002). Peripheral areas are most limited in terms of development, as a consequence of their remoteness from centres of influence and the fact that borders have frequently formed in areas of natural barriers. It is precisely these natural barriers that significantly impede the more successful development of rural areas, since the majority of businesses and employment are located in just a few centres, while the periphery experiences economic stagnation.

Little has been done to date in the field of cross-cultural research with regard to Slovenia. Since the country did not have extensive contacts with foreign markets until a few years ago (with the exception of the former Yugoslavia, which in this case is hardly classified as a foreign market, in view of the powerful historical connections), the need for research of this kind was not very pressing. More recently, the most influenced and systematic cross-cultural study was conducted by Hofstede (2001), and shows that Slovenes score highly in the power distance dimension. They accept a hierarchical order in which everybody has a place and which needs no further justification. Slovenia could be described as a feminine society, where the focus is on “working in order to live” and on well-being. People living in this country have a high preference for avoiding uncertainty, their daily behaviour is perceived as very well-organised, hard-working, and precision and punctuality are the norms. Security is a crucial element in individual motivation.

## Importance of Bias Testing in the Survey’s Adaptation Process and Earlier Translations of CQS in Slovenia

The survey’s adaptation maximizes cultural appropriateness, and thereby minimizes bias. Adaptation amounts to a combination of close translation of the parts of the instrument that are assumed to be adequate in the target culture, such as test instructions and items, and an adjustment of other parts when a close translation would be inadequate for linguistic, cultural, or psychometric reasons (Hambleton & De Jong, 2003; Harkness, Mohler, & Van de Vijver, 2003). Malda et al. (2008) proposed five different types of adaptation for cognitive tests to help systemize the adaptation process and the choices made in this process. In the present CQS adaptation we took into account the language-driven adaptation (unavailability of semantically equivalent words across languages or from structural differences between languages), the cultural-driven adaptation (it results from different cultural norms, values, communication styles, customs, or practices), and the theory-driven adaptation (it involves changes that are required for theoretical reasons).

The present adaptation was motivated by the need to fit the cultural, social, and linguistic dimensions. Before the pilot study for the CQS among Slovene university students was conducted (Jakovljević, Kodrič, Prelog, & Tumpej, 2016), the CQS had already been translated twice into Slovene. The researchers used it to measure the connection between knowledge and cultural intelligence (Gotnik Urnaut, 2014), and to describe the role of cultural intelligence in creativity in a culturally diverse environment (Bogilović & Škerlavaj, 2016). In the Slovene pilot study (Jakovljević, Kodrič, Prelog, & Tumpej, 2016) participated 527 students (114 males and 413 females, with a mean age 22.79 years, *SD* = 3.89) from the three biggest universities in Slovenia. A majority of the sample had been abroad already (99.6%), 97.9% speak at least one foreign language on the communication level. We compared its results whit previous studies Slovene studies (Table 1). Reliability analysis revealed that the internal consistencies (Cronbach’s alphas) of all dimensions were acceptable (α > .70) to good (α > .80), and the CQS questionnaire is appropriate for further scientific use and development.

**Table 1:** Previous Slovene Studies - Means (M), Standard Deviations (SD), and Internal Consistencies (α) for Dimensions of CQS

CQS Dimension	Students of commercial science (*N* = 107)^a^			Employees in the Adriatic region (*N* = 787)^b^			Students in the pilot study (*N* = 527)^c^		
	*M*	*SD*	α	*M*	*SD*	α	*M*	*SD*	α
Metacognitive CQ	4.89	1.31	-	4.78	1.49	0.92	5.06	1.08	0.76
Cognitive CQ	3.82	0.97	-	4.33	1.33	0.87	3.94	1.00	0.81
Motivational CQ	5.25	1.19	-	4.71	1.48	0.91	4.78	1.07	0.74
Behavioral CQ	4.76	1.19	-	4.34	1.41	0.89	4.52	1.15	0.79
CQS	4.63	0.95	-	-	-	-	4.52	0.88	0.90

^a^Gotnik Urnaut, 2014. ^b^Bogilović & Škerlavaj, 2016. ^c^Jakovljević, Kodrič, Prelog, & Tumpej, 2016.

To date many guidelines for test adaptation have been proposed, but there is still no agreement about standards to follow. We conceptualise our study by applying a systematic procedure (Mohler, Dorer, de Jong, & Hu, 2016) for cross-cultural scale adaptation. To avoid misunderstanding of some culturally based concepts that can threaten the validity of intergroup comparisons (Van de Vijver & Hambleton, 1996) we also focused on item bias in the CQS. Item bias refers to item-specific problems in cross-cultural comparisons, such as item ambiguity due to poor item translations or culture-specific elements (Malda et al., 2008).

## Aim and Research Questions

The aim of the qualitative research (implementation of focus groups) was to establish the content validity of the Cultural Intelligence Scale and adapt it to the Slovenian population. The translation and the adaptation of the CQS were conducted through two separate international teams collaborating and working closely together. The Slovenian-American group of researchers set the following objectives:

1. To describe general comprehension of the CQS on the basis of the responses and reactions of participants and further discussion among a Slovenian public.
2. To establish the level of understanding, identify barriers in the perception of individual questions among Slovenes, and recognise the characteristics of their attitude towards the topic of cross-cultural interaction.
3. To determine a suitable approach in order to supplement and shape the final translation of individual items, expressions and definitions in the Slovenian version of the CQS.

## Method

### Participants

Two focus groups were conducted. The participants in the first group (Table 2) were inhabitants of the capital city, Ljubljana (urban environment), while the second group (Table 3) consisted of individuals from Koper (border region, rural area). All participants were university graduates, were in employment and were ethnic Slovenes.

**Table 2:** Demographic Characteristics of Participants From the Urban Environment

Gender	Age	Employment	Marital status
Male	47	Business consultant/economist	Cohabiting
Male	32	Instructor working in an old people's home	Single
Female	35	Adviser in commercial activities	Cohabiting
Female	55	Development	Married
Female	46	Administrative work	Cohabiting
Male	31	Surveyor, computer operator	Cohabiting
Male	51	Brokerage, commerce, consultancy	Divorced
Male	42	Teacher	Cohabiting
Female	44	Not employed	Cohabiting
Female	52	Slovene language teacher, librarian	Divorced

**Table 3:** Demographic Characteristics of Participants From the Rural Environment

Gender	Age	Employment	Marital status
Female	49	Consolidated financial statements	Married
Female	48	Accounts department	Cohabiting
Male	35	Bank employee	Cohabiting
Male	33	Banking consultant	Cohabiting
Male	41	Accountant	Single
Female	28	University professor	Cohabiting
Female	37	Healthcare engineer	Cohabiting
Female	44	Tax administrator	Married
Female	29	Administration/accounts	Single

### Assessment and Measures

The self-reported *Cultural Intelligence Scale* (CQS; Ang et al., 2007), was used to measure person’s ability to function effectively in culturally diverse situations. The CQS measures four primary factors, which represent distinct cultural intelligence capabilities – drive, knowledge, strategy, and action. With 20 items it measures four dimensions of cultural intelligence: four items for metacognitive CQ (e. g., I am conscious of the cultural knowledge I use when interacting with people with different cultural backgrounds.), six items for cognitive CQ (e. g., I know the legal and economic systems of other cultures.), five items for motivational CQ (e. g., I enjoy interacting with people from different cultures.), and five items for behavioral CQ (e. g., I change my verbal behavior when a cross-cultural interaction requires it.). All items are scored on the seven-point rating scale, ranging from 1 (strongly disagree) to 7 (strongly agree). A good psychometric stability of the CQS across samples, time, counties, and methods has been provided (Ang, Van Dyne, & Tan, 2012).

*Focus groups* represent a qualitative method of data collection and fall among non-standardised techniques that are not highly structured (Kitzinger, 1995). They are also widely used in cross-cultural psychology that also require interaction between the researcher and cultural informants (Smith, Fischer, Vignoles, & Bond, 2013). They give answers to the questions what, how and why, but not how much, and attention is focused on the mental process that takes place in the participants when they discuss and reflect on the topic of discussion (Morgan, 1997). They can be used to discover views, opinions and justifications regarding certain phenomena. For this reason, focus groups serve to give deep insight into the research problem. Participants can be chosen on the basis of a common characteristic connected to the topic of the focus groups or the research question (Flick, 1998; Krueger & Casey, 2000; Willig, 2001). In the case of this research the characteristic is their belonging either to the urban group or to the rural group.

As Mohler et al. (2016) suggests, in the phase of reviewing the translated survey we need to check suggestions made by the adaptation team with groups formed from other locations and adjust the adaptation proposals accordingly. The focus group method was chosen because discussion helps to define understanding of the issue of cross-cultural interaction among participants, and also allows to discover their interpretations of the items in the CQS. At the same time, however, within the groups, participants formulate answers on the basis of their own personal convictions, and also on the basis of dominant cultural standards and models.

### Procedure

The focus of our research was the implementation of two focused groups. To begin with, a moderator asked the participants to complete the CQS, and then explained to them the aim of our research and began the discussion. The procedure took place in this order so that spontaneous answers to the questions in the scale from the participants could be obtained before they were aware of the aim of the research.

The discussion began with the moderator asking the participants to try and explain the importance of cross-cultural communication and define its benefits. The participants returned to an exchange of opinions on this topic later on in the discussion. Their observations and answers helped highlight the arguments, justifications and positions that served as a basis for the later adaptation of individual scale items.

Both focus groups were recorded (video and audio), and the material obtained later served in the production of a transcript of the discussion. In writing up the transcript a code was used to indicate which of the participants was responsible for an individual quotation. After this all the quotations relating to a specific item were grouped, and the quotations of both groups were combined. The next step consisted of a content analysis based on the data obtained in the focused groups. The observers’ notes were used as the *sampling unit* (Krippendorff, 1980) and the missing information was later added by listening to the recordings. *Units of analysis* are the smallest units of content referring to a specific topic. They include experience and ideas that the participants described during the discussion about the individual item. *The unit of context* usually refers to several units of analysis; in our study, the thematic sequence of an individual dimension (metacognitive, cognitive, motivational and behavioural) was used as a unit of context.

In the final step the documentation of changes and their rationale was made available by authors. The documentation taken by the research team forms a basis for the use of the CQS in the future, as Mohler et al. (2016) suggested.

## Results

Generally speaking, the response to the CQS was good. The participants from the urban environment had a more positive opinion and felt that the scale was not too difficult, but that completing it required a considerable amount of thought. They felt that it was suitable for all levels of education and all ages, but that previous contact with other cultures was a precondition for being able to complete the scale. The participants from the rural environment pointed out that some of the questions were difficult. During the discussion it was possible to observe that the latter participants were less familiar with this topic and had not yet formed a clear opinion about it. As a consequence, they also found it more difficult to complete the scale.

Both groups noticed that some of the questions were very similar or were repeated with slightly different wording, while the meaning remained the same. They themselves said that this was probably to do with the psychological method of asking the questions.

### Metacognitive Dimension

The participants took most time over this unit. It was not clear to them what the aim of the items was, or what the authors of the scale wanted to find out from them. The items seemed quite complex to them and they said that they would like them to be simplified. They were also of the opinion that the items in this unit are repeated: that they refer to the same things but in different words.

The participants thought that the first item ("I am conscious of the cultural knowledge I use when interacting with people with different cultural backgrounds") was unnecessary. They saw this sentence as broad, too general, long and relative. They compared it to the third item ("I am conscious of the cultural knowledge I apply to cross-cultural interactions"), which they considered to be identical or very similar, for which reason they felt it did not make sense to use both. Subsequent examination revealed that the participants had answered those questions in the same or a very similar way.

The item "I adjust my cultural knowledge as I interact with people from a culture that is unfamiliar to me" appeared contradictory to the participants. If we are talking about an unfamiliar culture, this means that a person do not have knowledge about it, and consequently cannot adjust it. They agreed that it would be more suitable to say "When I interact with people from a culture with which I am unfamiliar, I try to adjust my behaviour, conduct, knowledge".

The participants said they would like to see more consistency in the phrases used in the individual items. This is a reference to the phrases "interact with people from a culture that is unfamiliar to me", "interacting with people with different cultural backgrounds", "cross-cultural interactions". In all cases, they understood this to be talking about the same thing but were bothered by the fact that the phrases are subtly different and thus give the impression that they are referring to something else. As one participant from the focus group said, this was presumably a psychological method of putting statements, where they ask the same thing in several different ways – which provokes resistance among the participants.

Regarding the item "I check the accuracy of my cultural knowledge as I interact with people from different cultures", the participants stated that an individual does not check his or her cultural knowledge. Rather, this is an automatic process that happens inside us, in every individual, and not out loud. This checking thus does not take place at the conscious level, but unconsciously. For the same reason the participants objected to the phrase "I am conscious of the [cultural] knowledge" and wished to replace it with, for example, "I apply [cultural] knowledge."

Participants compared the phrase "different cultural backgrounds" to the phrase "other cultural backgrounds". "Different" can have a negative connotation, so they agreed that the expression "other cultural backgrounds" was more suitable.

### Cognitive Dimension

This set of items was clear and comprehensible to the participants and easier to answer. They gave a large number of examples for each item and knew exactly what the author of the scale wanted to find out from them and what was important in an individual item. Since understanding was better, this discussion unit took the least time.

In all the items from this unit, the participants were bothered by the phrase "*I know* [specific characteristics] *of other cultures*", which suggests that the respondent should know all the characteristics of all other cultures and not only of specific, individual cultures. The statement "I know the legal and economic systems of other cultures" seems excessively self-confident for a Slovenian public. Such an entire category causes many individuals to fear that they are accepting responsibility and self-confidently assessing their own knowledge, since this phrase implies an absolute category; in this case, knowledge of all the characteristics of all cultures. The participants therefore proposed amending the phrase to read "I know *specific* characteristics of *some* other cultures", which would be easier for them to fulfil.

In the case of the item "I know the marriage systems of other cultures", it would be more suitable to say "I know the marriage customs". This opinion was expressed by the majority of participants, while a participant who has a background in Slavic languages added – citing the reference dictionary of standard literary Slovene.

When discussing legal and economic systems, language and art, the association is very frequently associated with countries rather than with cultures themselves. The participants therefore wondered whether it would be a good idea in this case to replace the word "cultures" with the word "countries". They discussed the laws of a country, the economy and the language that is present in a specific country or nation.

The phrase "cultural values and religious beliefs of other cultures" bothered them since the adjective "cultural" seemed superfluous. When talking about the values of other cultures, it is enough simply to say "I know the values of other cultures", since all values are in this case cultural. The word "cultural" can also appear superfluous in that the emphasis on the word "cultural" narrows its meaning, with the result that the individual more frequently thinks merely about cultural forms such as art, music, etc., which are the usual associations with the phrase "cultural values".

Once again, the participants expressed the desire that the item "I know the rules for expressing non-verbal behaviours *in other cultures*" should be made consistent with those items reading "I know [specific characteristics] *of other cultures*". In other words, instead of the phrase "in other cultures", to simply use "of other cultures" and in this way avoid additionally burdening the mental capacities with new phrases which mean the same thing.

### Motivational Dimension

The third unit or set of items was well understood by the participants. The items seemed sufficiently concrete to the individual participants, who were therefore able to give some examples for each sentence. They did, however, propose some improvements.

The sentence "I am confident that I can socialise with locals in a culture that is unfamiliar to me" was translated as "*Pri socialnih stikih z ljudmi iz meni neznanih kultur sem samozavesten*" [literally: "I am confident in social interactions with people from cultures unfamiliar to me"]. The participants felt that the word *socialnih* (social) was unnecessary here, since it could be associated with social networks or an individual's online interactions, which gives an entirely different view of the situation. They all agreed that it would be better to leave this word out.

The sentence "I am sure I can deal with the stresses of adjusting to a culture that is new to me" was considered by the participants to be too long. They felt that the phrase "deal with the stresses" was inappropriate, too strong and somewhat negative. They proposed rephrasing the sentence to read "I am capable of adjusting" or "I am able to adjust". They also pointed out here that another critical factor is whether the individual is willing to adjust. They gave numerous examples of where individuals are capable of adjusting but do not wish to, a situation in which the participants see a more significant problem.

The sentence "I enjoy living in cultures that are unfamiliar to me" was in their view unsuitably formulated. If a culture is unfamiliar to you, you cannot be living there; the sentence is therefore contradictory. You cannot know whether you enjoy living in a culture that is unfamiliar to you because you are not familiar with it and do not know what it is like to live there. You can only know this once you are already living there. Not only that, but the concept seemed a little "cowboyish", as one participant from the first focus group put it, "*as though this happens goodness knows how many times, from one day to the next*." They proposed several alternative ways of formulating the sentence, for example: "I enjoy travelling to unfamiliar places", "I enjoy exploring", "I enjoy getting to know", "I would like to live in", "I would be willing to live in cultures that are unfamiliar to me".

The sentence "I am confident that I can get accustomed to the shopping conditions in a different culture" was considered by the participants to be adequately comprehensible. They did, however, point out a stylistic incongruity in the Slovene translation, since the word/root form *navad* appears twice in the sentence. It would be grammatically better to replace the word *navadim* ("get accustomed to") with a different word meaning "adjust to" or "accept".

### Behavioral Dimension

The last set of items caused more confusion in the participants than the previous two. Again, it was not entirely clear what specific items were asking, while in the participants' opinion the meaning of some items overlapped.

The word "cross-cultural" (interaction) was translated as *večkulturna* (literally: "multicultural"). In their opinion it would be better to replace this with the word *medkulturna* (literally: "intercultural"). "Multicultural" assumes that a large number of cultures are involved. The participants wondered how many cultures were meant by *večkulturna*. As one of the participants put it: "*there must be at least five of them*". "Intercultural", on the other hand, merely assumes that there are two or more cultures.

With regard to the item "I use pause and silence differently to suit different cross-cultural situations", the participants wondered about the difference between pause and silence. They discussed the meaning of each of the words and it turned out that each participant had his or her conception of what they mean. Owing to the difficulty of distinguishing between them, it was proposed that only one should be used. They also drew attention to the use of the plural in the Slovene translation of this sentence (literally: "Use of pauses and silences"), which in this case is unnecessary and it would be more appropriate to use the singular.

The item "I vary the rate of my speaking when a cross-cultural situation requires it" was considered unnecessary, since its meaning is also covered by the earlier item "I change my verbal behaviour", where the rate of speaking is merely one type of verbal behaviour.

In the case of the item "I change my non-verbal behaviour when a cross-cultural situation requires it", the participants pointed out that this seemed to them to be the same as "I know the rules for expressing non-verbal behaviours", a fact that demonstrates that they do not distinguish between knowledge (cognitive unit) and behaviour (behavioural unit). They unanimously agreed that the two items were asking the same thing, meaning that one of them should be omitted.

They saw the item "I alter my facial expressions when a cross-cultural interaction requires it" as part of the item "I change my non-verbal behaviour when a cross-cultural situation requires it", since changing facial expressions is part of non-verbal communication. They felt it would be more suitable to use the Slovene expression *obrazni izraz* (facial expression) than the current *izraz na obrazu* (literally: "expression on the face"). Since we are constantly *changing* our facial expressions (not only in cross-cultural situations), the participants felt it would be better to say "I adjust my facial expressions" – given that adjustment is something that is important in a cross-cultural situation.

### Definitions of CQS by Slovenian Participants

At the end of the discussion of the items, the moderator asked each participant to give their definition of cultural intelligence. Their answers were interesting and diverse, since each of the participants tried to add something of their own and to present their own view. Even so, it was evident that this worked well in the first group, while in the second group (rural area) the opinions of the individuals appeared to be conditioned by the opinions of the other members who had already given a definition before them (something already evident during the earlier discussion). It turned out that each participant in the first group had their own view or an already formed opinion about the topic, while in the second group the majority of individuals had never thought about the topic at a conscious level before this discussion – this was effectively their first contact with it – and were therefore more easily swayed by the opinions of others. Their group behaviour on some points can be described as typical for nations with a high score in the power distance dimension (Hofstede, 2001) where subordinates expect to be told what to do, or in our study what to think about the topic of cultural intelligence. It could be observed during the discussion that the participants from the urban focus group were aware that they were more in contact with different cultures that they lived in a more intercultural environment in comparison to the participants from the rural environment. One man from the rural environment made the following comment about the importance of cultural intelligence: "*It depends on where you live. If it's a multicultural environment, you need it, but if you live somewhere in Koper it is rarely an issue.*" Meanwhile, a participant from the urban environment commented: "*The fact is, we live in an intercultural environment*."

Similar themes emerged in both focus groups. The participants began by pointing out that cultural intelligence is about knowledge of one's own and other cultures and awareness of the existence of other cultures, which overlaps with the first and second dimensions of the scale. They also drew attention to the importance of knowledge of foreign cultures, while at the same time knowledge of one's own culture represents the basis for getting to know a foreign culture. As a participant from the rural group put it: "*This is one stage of knowledge of the individual – knowledge of the habits, customs, the historical background of one's own country, and then understanding and adjustment to other countries*." Those participants who described cultural intelligence as knowledge believe that it can be changed and acquired, depending on how much the individual is prepared to learn. We can acquire it in various fields: "*...from television, in newspapers and magazines, in the library, at the theatre, in all cultural institutions*." In connection with knowledge, familiarity and behaviour, a male participant from the first group described cultural intelligence as a ladder: "*I see cultural intelligence as a ladder. If we know, accept and understand, we are higher up this ladder of cultural intelligence*."

The most common topic, present in the majority of the definitions provided by the participants, was cultural intelligence as a willingness to accept the foreign. A man from the first group neatly described this: "*The fact is, we live in an intercultural society, this is something we must admit. It depends on us how we accept this*." Participants highlighted as extremely important the personal view that each individual has of foreign cultures, the attitude towards the foreign and a positive attitude towards others: "*...it's about having a positive attitude towards other cultures and a willingness and openness to get to know other cultures*." This, for them, represents the foundation of cultural intelligence. Without this positive basis, the elements that follow on from this are not possible. It is therefore not so much a question of the individual's abilities and knowledge, but of attitude. They underlined the importance of being tolerant, of respecting what is foreign, of desiring to get to know and adapt to and finally accept the foreign: "*...a curiosity, a desire to get to know new, different, other cultures. Moreover, the desire and openness to adapt to others.*"

They see the basis for a positive attitude towards other cultures as having, in the first place, a positive attitude towards your own culture, since this enables you to have a positive attitude towards other cultures and at the same time to maintain a balance, so that other cultures do not become dominant. This can be illustrated by the statement "*Love your own and respect the foreign.*" One participant from the urban environment made the following comment about cultural intelligence: "[The culturally intelligent person] *will not allow themselves to be overwhelmed by another culture. They will always find a consensus and seek compromises – just as happens today in society, so that we can live*." A positive attitude towards others, respect and tolerance also require a certain degree of emotional intelligence, which allows you to cultivate positive feelings towards others. It could also be described as a feminine society (Hofstede, 2001), where people strive for consensus, value equality and solidarity. Thus, the participants also mentioned emotional intelligence as one of the conditions for cultural intelligence.

The category that appeared most frequently was that of the willingness and desire to accept and adapt to the foreign, which coincides in the scale with the unit containing motivational items. As can be seen from the answers of individuals, this dimension of the scale represents for them the foundation of an individual's cultural intelligence and the basis for all the other units (the individual's awareness, knowledge and behaviour).

## Discussion

### Limitations of the Study and Recommendations for Further Research

The findings obtained indicate the need for specific changes to the CQS in the event of it being used for a Slovenian public. Despite the small sample size and the two focus groups, the findings can be used to develop hypotheses for a large-scale national survey. They also show a good overview of the definitions of cultural intelligence offered by the participants – what cross-cultural interaction and cultural intelligence mean to them, and what attitude they have towards the latter – which gives an insight into the way people think about the concept itself. The next step could be a Slovenia-wide study using an adapted CQS, followed by analysis of how the results differ by region, gender, education and age of respondents.

The context in which the present focus groups were run was also special from the sociological point of view, since this was a period in which a large number of migrants from the Middle East were arriving in and passing through Slovenia. As a result, it was possible to sense a universal belief that it is necessary to accept other nations, but at the same time a general (media-fuelled) fear of the cultures that these refugees bring. Their answers confirmed Hofstede’s findings (2001) about the high uncertainty avoidance result in this country, which could be perceived as rigid codes of belief and behaviour, and also the need for personal security. Terrorism was also mentioned a few times, and it was apparent that this topic was actively present "in the air", although the participants tried to avoid it.

Perhaps a consequence of this is the underlining (by some of the participants) of the importance of knowing which foreigners and other nations we are talking about, where it was possible to sense slightly more respect and consideration for northern cultures (e.g. Germans and Scandinavians) in comparison to those foreigners who come to Slovenia to seek a better life (the countries of the former Yugoslavia, refugees from the Middle East).

It is also recommended that the same research be conducted in the other former Yugoslav republics (e.g. Bosnia), which would enable appropriate comparison. This is specifically relevant due to the previously mentioned historical connection of these countries, where Slovenia used to be in the same country with them and therefore shares some of their cultures, but on the other hand, there is much more “western” influence present in this small country, because of its connection with the German sphere to the north and Italy to the west, which enabled Slovenia to be more developed than other Yugoslav republics.

Understanding of the CQS and the individual items therein by the participants was influenced by various geographical, ideological, social, cultural and psychological factors, which may have acted as motivators or as barriers to active participation in this study and the further presentation of cultural practices and predispositions. The participants' understanding of the scale was interesting, sometimes surprising, and represented a mixture of their psychological, cognitive and cultural responses.

They see some barriers also. The perceived difficulty of some of the items, particularly in the metacognitive dimension, where the participants could not entirely understand what kind of metacognitive processes were involved. They had doubts about the logic of the items themselves, since, as they pointed out, awareness involves automatic, subconscious processes, which made it hard for them to think about them and rank them on a scale of 1 to 7. A further difficulty was that they did not have a clear idea of what the authors of the scale wanted to find out from them in this dimension, or what the goal was. Similarly, the fact that the metacognitive dimension appears at the beginning makes their work even more difficult. It would therefore be easier to place the dimension later, once individuals are already a little more familiar with the scale and the topic.

The apparently large number of items which participants considered to have the same or a similar meaning. They claimed that phrases were slightly altered or placed in a different order to give the impression that they were referring to something else. This caused resistance among participants and the feeling that this was a "psychological method of asking questions". Their attitude to this was somewhat negative, since they felt that the same thing was being asked in several different ways. They also felt that the content of some items was already included in other items. A desire to simplify the CQS is obviously evident here. As the participants themselves pointed out, the scale is difficult and long – a judgement that reflects their negative attitude towards the structure of the scale. They would prefer a transparent, shorter and more efficient structure, since the average individual from the Slovenian public is not used to thinking in such depth about a specific issue or problem. The scale undoubtedly surpassed their usual quantity of rational reflection on such topics. This was particularly evident in the case of the group from the rural environment.

The phrase "I know [e.g. the legal systems] of other cultures" seems excessively confident to Slovenian participants. Such an absolute category causes many individuals to fear that they are accepting responsibility and self-confidently assessing their own knowledge, since this phrase implies an absolute category; in this case, knowledge of all the characteristics of all cultures. This may be a consequence of modesty – defined as self-restraint from excessive vanity that can possess moral and/or ethical dimensions – a cultural virtue that is strongly present among Slovenes (71.6% of Slovenia’s population in 1991 were Roman Catholic, and 57.8% in 2002; SURS, 2002), and the reason why they feel it would be more appropriate to slightly tone down such absolute expressions. Gradišek (2014) confirmed this in the study of Slovene teachers’ virtues, where modesty was placed higher compared to the USA or Switzerland (Park, Peterson, & Seligman, 2006).

Highlighting the importance of the individual's willingness and desire to adapt to other cultures. Individuals perceive that this is the basis for all other processes described by the behavioural and metacognitive dimensions. If an individual does not have this willingness, the other components are unimportant. The strong emphasis on the latter is the consequence of the participants' frequent interactions with individuals who have immigrated from the countries of the former Yugoslavia, whom they perceive as capable and culturally intelligent, yet in whom this willingness does not find expression, since their lack of motivation to adapt is a more powerful stimulator. Among such individuals (e.g. those from the countries of the former Yugoslavia), cultural adaptation is perceived as unnecessary, since they join together in large groups, form a subculture and maintain their language – which Slovenes can understand. For these reasons, they are not willing to adapt.

These listed suggestions – the language-driven (mostly observed in all four dimensions), the culture-driven (mostly found in the cognitive dimension and in the motivational dimension), and the theory-driven (presented mainly in the metacognitive dimension and in the behaviour dimension) observations presented valuable information for the preparation of the final version of CQS. The language-driven adaptation was based on many different suggestions and solutions given by the participants, and it required elaborate piloting to evaluate the success of adaptation. On the other hand, the theory-driven adaptation was more focused and less responsive to disagreement based on widely investigated and documented bases.

The present qualitative research has helped define specific aspects within the perception of the CQS using a Slovene sample. A good understanding of the cultural context of the target version is crucial for adapting multicultural surveys. Barriers to perception were identified, and the specific characteristics of the Slovene public's understanding of the scale in connection with cross-cultural interaction were defined. Moreover, it seems encouraging that the importance and suitability of the research topic were readily accepted by all the participants.
